# A gendered network analysis of somatic, psychological, and healthcare utilization patterns among parents of service members in wartime

**DOI:** 10.3389/fpubh.2026.1742802

**Published:** 2026-03-27

**Authors:** Yaira Hamama-Raz, Liat Hamama

**Affiliations:** 1 School of Social Work, Ariel University, Ariel, Israel; 2 Bob Shapell School of Social Work, Tel Aviv University, Tel Aviv-Yafo, Israel

**Keywords:** health status, military personnel, network analysis, parents, sex factors, war

## Abstract

**Introduction:**

Parents of actively serving soldiers experience sustained stress that affects psychological, somatic, and behavioral health, yet the interrelations among these domains and gender differences are understudied.

**Methods:**

We studied 402 Israeli parents (201 mothers, 201 fathers from unrelated households) of sons/daughters aged 18–27 serving in regular or reserve duty during wartime, recruited via a national online panel (data collected in August 2025). Participants completed self-report questionnaires related to psychological, somatic, and behavioral health domains. Network analysis was conducted to observe gendered network differences between mothers and fathers, comparing them via edge and centrality invariance tests, and evaluating them using global architecture, bridge centrality, predictability, and small-world properties.

**Results:**

Mothers reported greater psychological distress, sleep disturbances, somatic symptoms, pain, and more frequent healthcare utilization. Their network had shorter path lengths, higher predictability, and healthcare visits as a dominant bridge; somatic symptoms and sleep disturbances were key connectors. Fathers' network was denser yet more compartmentalized, with bridges concentrated on somatic/pain and adverse events; alcohol use was isolated.

**Conclusions:**

Mothers exhibited more integrated, rapidly spreading symptom networks closely tied to healthcare engagement, whereas fathers' networks were more segmented and less visible in terms of help-seeking. These patterns support gender-sensitive screening and interventions for parents of service members.

## Introduction

Military service, whether mandatory or reserve, constitutes a distinctive sociocultural context whose effects extend beyond service members to their families (1–4). Parents in particular face sustained stress as they navigate their offspring's service and potential exposure to combat (2, 3, 5–7). Prior research indicates that family members of soldiers may experience distress and secondary traumatization, including elevated anxiety, posttraumatic symptoms, sleep disruption, heightened somatic symptoms, and increased engagement with healthcare services (2, 5–9). Likewise, evidence from caregiving contexts (e.g., pediatric chronic illness) documents poorer parental health outcomes under sustained stress (10), and intolerance of uncertainty has been implicated in anxiety, depression, and maladaptive coping (11–13). Consistent with these patterns, a recent study of Israeli combat veterans and their parents indicated family-level transmission of stress, mediated by parental accommodation and influenced by lower distress tolerance (3).

In the present study, we focused on Israeli mothers and fathers from unrelated households whose sons or daughters were actively serving (regular or reserve) during wartime, examining differences both within and across parental groups. Prior research has indicated that mothers report higher distress and worry than fathers during wartime (2, 5, 7) and that parents exposed to ongoing war and prolonged armed conflict exhibit increased rates of depression, anxiety, and emotional distress, with mothers particularly affected (14). However, these studies have primarily examined differences in psychological burden, whereas somatic symptoms, health behaviors, and healthcare utilization during wartime have been rarely assessed. More broadly, previous studies have shown that women tend to seek more health-related information (50) and to utilize primary care more frequently for both physical and mental health concerns than men (15). Such disparities are thought to be shaped more by socialized gender roles than by underlying physiology, channeling men and women into different exposures to stressful life events and, consequently, different vulnerabilities to health issues (16).

In this study, we distinguish sex (the classification of individuals as male or female; mothers vs. fathers) on the basis of biological and demographic criteria, from gender, understood as the socially constructed roles, behaviors, expectations, and norms associated with being a mother or a father. These gendered norms influence how distress is expressed, managed, and reflected across health domains.

To investigate gender-related differences across health domains among parents of actively serving soldiers, we applied network methods. This approach maps interrelations among health symptoms, health behaviors, and healthcare utilization, including psychological distress [reflected in symptoms of depression and anxiety; (17)], intolerance of uncertainty [the tendency to perceive uncertainty as stressful or threatening and to respond with cognitive, emotional, and behavioral reactions; (12)], somatic symptoms defined in this study as subjectively experienced bodily complaints [e.g., headaches, gastrointestinal discomfort, fatigue; (18)], reflecting embodied manifestations of stress rather than medically diagnosed conditions, sleep disturbances, pain (type and severity), medication use, smoking and alcohol use, and healthcare utilization (visits and frequency). By capturing relations among variables rather than focusing solely on group means, network analysis reveals how health domains are organized and interrelated within each group.

## Methods

### Participants

The study included 402 parents (201 fathers and 201 mothers) who were not from the same families. Fathers were older than mothers on average (53.5 ± 4.7 vs. 51.3 ± 5.1 years, *q* < 0.001). Most participants were married or in a relationship (80%), with a mean number of three children and similar years of education (M =15 years for both). Economic status was predominantly moderate to good. Self-rated health before the war was largely good/very good (≈91% in both groups). During the ongoing war, health ratings shifted downward, with mothers more often reporting “not so good” health and fewer “very good” ratings compared to fathers (*q* = 0.067). Prior use of mental health services was relatively low but reported higher among mothers (16% vs. 10%, *p* = 0.3). Regarding the military service of the parents' offspring, most families had one child in military service; the majority were serving as regular duty soldiers, with about one-quarter serving in the reserves. One-fifth reported an offspring serving in an active war zone. Reports of injuries during the offspring's service were uncommon and similar across groups, with mental injuries reported more often than physical injuries (see Table 1).

**Table 1:** Fathers' and mothers' characteristics.

Characteristics	Fathers, *N* = 201^a^	Mothers, *N* = 201^a^	*p*-value^b^	*q*-value^c^
Age			< 0.001	< 0.001
	53.54 (4.69)	51.34 (5.12)
Family status			0.003	0.021
Married or in a relationship	158 (79%)	163 (81%)		
Separated/divorced	34 (17%)	31 (15%)		
Single	4 (2.0%)	3 (1.5%)		
Widowed	5 (2.5%)	4 (2.0%)		
Number of children			>0.9	>0.9
	3.00 (2.00, 3.00)	3.00 (2.00, 4.00)
Years of education			0.4	0.6
	15.00 (14.00, 17.00)	15.00 (12.00, 17.00)
Economic status			0.3	0.5
Bad	1 (0.5%)	0 (0%)		
Not good	14 (7.0%)	18 (9.0%)		
Moderate	83 (41%)	98 (49%)		
Good	83 (41%)	72 (36%)		
Very good	20 (10.0%)	13 (6.5%)		
Health before the war			>0.9	>0.9
Not so good	18 (9.0%)	16 (8.0%)		
Good	147 (73%)	147 (73%)		
Very good	36 (18%)	38 (19%)		
Health during the ongoing war			0.013	0.067
Bad	4 (2.0%)	8 (4.0%)		
Not so good	58 (29%)	75 (37%)		
Good	118 (59%)	111 (55%)		
Very good	21 (10%)	7 (3.5%)		
Utilization of mental health services before the war			0.082	0.3
No	181 (90%)	169 (84%)		
Yes	20 (10.0%)	32 (16%)		
Number of offspring in military service			0.14	0.4
1	144 (72%)	152 (76%)		
2	53 (26%)	41 (20%)		
3	3 (1.5%)	8 (4.0%)		
4	1 (0.5%)	0 (0%)		
Military service type			>0.9	>0.9
Regular duty	151 (75%)	152 (76%)		
Reserve duty	50 (25%)	49 (24%)		
Serving in an active war zone			0.5	0.8
Yes	49 (24%)	40 (20%)		
No	131 (65%)	142 (71%)		
Unknown	21 (10%)	19 (9.5%)		
Injury during active military service			0.7	>0.9
Not injured	181 (90%)	181 (90%)		
Physically injured	5 (2.5%)	2 (1.0%)		
Mentally injured	11 (5.5%)	14 (7.0%)		
Both physically and mentally injured	4 (2.0%)	4 (2.0%)		
Don't know	181 (90%)	181 (90%)		

^a^Mean (SD); *n* (%); Median (Q1, Q3).

^b^Wilcoxon rank sum test; Fisher's exact test; Pearson's Chi-squared test.

^c^False discovery rate correction for multiple testing.

### Measures

Participants completed standardized self-report questionnaires, all of which had previously been employed among Israeli populations and had demonstrated robust psychometric properties.

*Sociodemographic characteristics* included participants' gender, age, marital status, education level, and religiosity. Participants also reported on their son's or daughter's military service: service type (regular or reserve), length of service, role (combat or non-combat), whether the soldier was permitted or able to share details of their service, and whether the soldier had sustained an injury and, if so, its type (mental, physical, or both).

*Traumatic life events* were assessed using the List of Threatening Experiences Questionnaire [LTE-Q; (19)], which assesses 12 severe adverse events experienced in the past 2 months (e.g., serious illness) with yes/no responses. The LTE-Q has previously been used in Israeli samples and demonstrated acceptable psychometric properties (20). The original questionnaire demonstrated Cronbach's alpha of 0.84 (19). In the current sample, Cronbach's alpha was α = 0.58. In addition, participants answered seven yes/no items about events related to October 7th, 2023, and the ensuing Swords of Iron War (e.g., loss of a family member or close friend, injury to a family member or close friend, and evacuation from home). For this 7-item index, total scores were computed as the sum of “yes” responses (range: 0–7). In this sample, McDonald's ω was 0.71.

*Intolerance of Uncertainty* was measured using the 12-item Intolerance of Uncertainty Scale [IUS-12; (21)], a short form of the original 27-item scale (22) that assesses one's tendency to find the possibility of negative events unacceptable, regardless of their likelihood (e.g., “It frustrates me not having all the information I need.”). Participants rated each item from 1 (“*not at all characteristic of me*”) to 5 (“*entirely characteristic of me*”). The IUS-12 has two subscales of uncertainty: prospective anxiety and inhibitory anxiety. A total score was computed by summing all items (range: 12–60), with higher scores indicating greater intolerance of uncertainty. The IUS-12 has demonstrated good psychometric properties in clinical and non-clinical samples (23) and has also been validated in Israeli samples (24). Cronbach's alpha in the current sample was α = 0.93.

*Psychological distress* was assessed using the 4-item Patient Health Questionnaire [PHQ-4; (17)], comprising a 2-item depression scale (e.g., “Feeling down, depressed, or hopeless”) and a 2-item anxiety scale (e.g., “Feeling nervous, anxious, or on edge”). Participants rated how often they were bothered by each problem over the past 2 weeks on a 4-point scale from 0 (“*not at all*”) to 3 (“*nearly every day*”). Total distress scores were calculated by summing all four items (range = 0–12) and categorized as normal (0–2), mild (3–5), moderate (6–8), or severe (9–12). Subscale scores were computed by summing the relevant two items; scores ≥3 on the anxiety items suggest clinically significant anxiety, and scores ≥3 on the depression items suggest clinically significant depression. In the original questionnaire, Cronbach's alphas were 0.85 for depression and 0.81 for anxiety (17). The Hebrew version of the PHQ-4 has demonstrated good reliability in Israeli populations (25). In the current sample, Cronbach's alpha for the total score was α = 0.88.

*Pain* was assessed via the Short-Form McGill Pain Questionnaire [SF-MPQ; (26)], a self-administered measure of pain quality and intensity. The SF-MPQ includes 15 descriptors assessing sensory (11 items, e.g., “hot/burning”) and affective (4 items; e.g., “fearful”) dimensions, each rated on a 4-point scale (0 = *no pain*, 1 = *mild*, 2 = *moderate*, 3 = *severe*). Sensory (range: 0–33) and affective (range: 0–12) scores were summed to yield a total score (range: 0–45), with higher scores indicating greater pain. The SF-MPQ is widely used in research and clinical settings and demonstrates good reliability, validity, and sensitivity to change (27). The Hebrew version of the SF-MPQ has demonstrated good reliability in Israeli populations (28). In the current sample, Cronbach's alpha for the total score was α = 0.93.

Additionally, participants completed two 0–10 visual analog scale (VAS) ratings: current physical pain intensity and affective pain, where 0 indicates “*no pain*” and 10 indicates “*worst possible pain*.” Mean (SD) scores were as follows: for physical pain intensity, M = 3.1(SD = 2.5) and for affective pain, M = 3.6 (SD = 2.8).

*Somatic symptoms* were measured using the Somatic Symptom Scale-8 [SSS-8; (18)]. Participants rated how much they were bothered by common somatic symptoms over the past seven days (e.g., “headaches”) on a 5-point scale from 0 (“*not at all*”) to 4 (“*very much*”). Total scores were computed by summing the eight items (range: 0–32), with higher scores indicating greater somatic symptom burden. Severity categories were divided into five categories (0–3 none/minimal, 4–7 low, 8–11 medium, 12–15 high, and 16–32 very high), and for this study, a cut-off of ≥12 indicated high somatic symptom severity. The Hebrew version of the SSS-8 has demonstrated good reliability in Israeli populations (28). Internal consistency (Cronbach's alpha) in the current sample was α = 0.86.

*Sleep disturbances* were assessed using the Jenkins Sleep Scale [JSS; (29)], a 4-item measure of common sleep difficulties over the past month (trouble falling asleep, waking several times per night, trouble staying asleep, and waking up tired). Each item is rated on a 6-point frequency scale: 0 = *not at all*, 1 = *up to 3 days a month*, 2 = *4–7 days a month*, 3 = *8–14 days a month*, 4 = *15–21 days a month*, and 5 = *22–31 days a month*. Total scores range from 0 to 20, with higher scores indicating more sleep problems. There is no universally accepted cutoff for poor sleep quality, although thresholds such as ≥12 have been proposed for elevated sleep problems [e.g., (30)]. For normative values, see Tibubos et al. (31). The Hebrew version of the JSS has demonstrated good reliability in Israeli populations (32). Internal consistency (Cronbach's alpha) in the current sample was α = 0.91.

### Procedure

The study was approved by the institutional review boards (IRBs) affiliated with each author. Following approval, participants were recruited through iPanel, Israel's largest online research platform, which adheres to ESOMAR international standards for research quality and participant management and includes approximately 100,000 respondents. Data were collected over 1 week (August 7–14, 2025). All participants were informed about the study's objectives and provided informed consent prior to participation.

Eligibility criteria required participants to be mothers or fathers of a son or daughter aged 18–27 who was actively serving in the military (regular or reserve) during wartime, to be fluent in reading Hebrew, and to provide informed consent. The questionnaire took approximately 12 min to complete. Respondents were instructed to refer consistently to a single child in active military service; if they had more than one child serving, they were asked to choose one and answer with that child in mind throughout.

### Data analysis

The analyses were conducted on 402 participants comprising 201 fathers and 201 mothers of offspring who were actively serving in the military (regular or reserve) during wartime. Importantly, the fathers and mothers were unrelated to each other, ensuring independence of observations between groups and allowing for clear comparison of paternal vs. maternal responses to their son's or daughter's active military service. Before the primary analyses, we examined the normal distribution of all main study measures using a series of Anderson-Darling normality tests. We also assessed the presence of multivariate outliers using the minimum variance estimator (MVE) approach with robust Mahalanobis distances (performed with the cov.rob function in R).

We found that all measures significantly deviated from normality (minimum A = 1.706, *p* = 0.0002). In addition, 93 observations were identified as multivariate outliers using the 97.5% χ^2^ cutoff criterion. Accordingly, we used robust statistics to ensure that our findings would not be unduly influenced by these departures from normality or the presence of outliers.

Additionally, 12.85% of the data were missing, with eight different patterns of missingness identified. Jamshidian and Jalal's non-parametric Missing Completely At Random (MCAR) test was conducted to examine the type of missingness. The test indicated that the data were missing at random (MAR) rather than completely at random, with Hawkin's test yielding a median χ^2^_(14)_ = 170.40, *p* = 5.69e-29, and the Anderson-Darling rank test showing a median T = 16.98, *p* = 4.95e-05. Both tests' significant results led us to reject the assumption that missingness was MCAR. Accordingly, we used multiple imputation (MI) with 13 datasets to handle missing data, using the micemd package with 10 iterations, ensuring that our analyses would appropriately account for the uncertainty introduced by the missing data patterns.

Participants were classified into two groups on the basis of their parental role: fathers (*n* = 201, 50%) and mothers (*n* = 201, 50%) of soldiers in active military service. The equal sample sizes between groups enhanced the statistical power and reliability of our comparative analyses. We began by examining group differences in somatic symptoms, health behaviors, and healthcare utilization using Yuen's robust *t*-tests for continuous variables and Fisher's exact tests for categorical variables, with false discovery rate (FDR) correction at 10% to control for multiple comparisons.

In the next step, we separately estimated two networks for the fathers' and mothers' groups. We specifically employed the extended Bayesian information criterion (EBIC) with graphical lasso (tuning parameter = 0.25), followed by refitting without the lasso penalty to obtain more accurate (non-shrunken) estimates of the partial correlations. The estimation process was performed with the estimateNetwork function of the bootnet R package. Following the estimation of the networks, we employed edges invariance tests with an FDR of 10% to control for the multiple analyses and centrality invariance tests.

In network analysis, *edges* represent partial correlations between two variables (known as nodes), revealing direct relationships after controlling for all other variables in the network. *Centrality* refers to a series of measures to evaluate the function of each node within the network, and centrality invariance tests allowed us to distinguish nodes that functioned differently between fathers' and mothers' networks. We focused on the following centrality scores: (i) Closeness—measuring how quickly information or influence can spread from a given node to other reachable nodes in the network; (ii) Betweenness—quantifying how often a node acts as a bridge along the shortest path between two other nodes, highlighting variables that may serve as crucial connectors in the coping and support systems; (iii) Strength—the sum of the weights of a node's edges, indicating the overall influence or importance of a variable in the network; and (iv) Expected Influence—a refined measure that accounts for both positive and negative connections, providing insight into a node's net impact on the network. The invariance tests were performed with the NetworkComparisonTest R package with 1,000 iterations.

To comprehensively evaluate network differences between fathers and mothers, we extended our analysis beyond traditional centrality measures. Network architecture was examined through global metrics including connectivity (mean absolute edge weights), global strength (sum of absolute edge weights), and density (proportion of possible edges present). These metrics offer insight into the organization of health domains (i.e., psychological, somatic, and behavioral alongside patterns of healthcare utilization) among mothers and fathers during the active military service of their offspring in wartime.

Given the anticipated community structures within networks, we conducted bridge centrality analysis using the networktools package. Bridge centrality measures NetworkComparisonTest R package including bridge strength, bridge betweenness, and bridge closeness—identify nodes that connect different psychological or behavioral domains within each parent group's network. These bridge nodes are particularly important as they represent potential intervention points that could support multiple aspects of parental wellbeing simultaneously.

The stability and reliability of our findings were assessed through edge weight stability analysis using bootstrap procedures with 1,000 iterations via the bootnet package. This analysis revealed which connections in fathers' and mothers' networks were estimated with high precision vs. those that showed greater variability, helping identify the most reliable targets for intervention.

To understand the deterministic nature of each network, we employed predictability analysis using the mgm package. This analysis estimates the proportion of variance in each node that can be explained by all other nodes in the network, revealing the extent to which each parent group's responses were interconnected vs. influenced by external factors not captured in the network.

Finally, we examined network topology through small-world analysis using the igraph package. Small-world properties, combining high local clustering with short average path lengths, indicated efficient information and support transmission within each parent group's network. Understanding these properties helped reveal how fathers and mothers differently organized health domains (i.e., psychological, somatic, and behavioral alongside patterns of healthcare utilization), and whether one group showed more adaptive network structures for managing the ongoing stress of having a son or daughter in active military service during wartime.

## Results

### Group differences between fathers and mothers

The results depicted in Table 2 highlight significant differences between fathers and mothers of soldiers in active military service across multiple health domains. After FDR correction, mothers demonstrated significantly higher levels of distress on nearly all measures that survived correction. Most notably, mothers reported substantially higher sleep disturbances (M = 2.83 vs. 2.19, *q* < 0.001) and psychological distress (M = 1.34 vs. 0.93, *q* < 0.001) than did fathers.

**Table 2:** Differences between fathers and mothers in study measures.

Characteristic	Fathers *N* = 201^a^	Mothers *N* = 201^a^	*p*-value^b^	*q*-value^c^
Adverse life events	1.09 (1.56)	1.16 (1.35)	0.11	0.2
Exposure to October 7th	0.89 (1.05)	0.95 (1.09)	0.6	0.6
Sleep disturbances	2.19 (1.49)	2.83 (1.43)	< 0.001	< 0.001
Psychological distress	0.93 (0.80)	1.34 (0.69)	< 0.001	< 0.001
Intolerance of uncertainty	2.50 (0.84)	2.80 (0.90)	0.001	0.002
Somatic symptoms	0.94 (0.79)	1.39 (0.81)	< 0.001	< 0.001
Healthcare visits	1.24 (0.99)	1.21 (0.86)	0.6	0.6
Healthcare frequency	2.15 (1.64)	2.34 (1.45)	0.011	0.021
Medication use	6.69 (2.15)	7.00 (2.33)	0.11	0.2
Smoking			0.13	0.2
No	175 (87%)	163 (81%)		
Yes	26 (13%)	38 (19%)		
Alcohol use			>0.9	>0.9
No	192 (96%)	193 (96%)		
Yes	9 (4.5%)	8 (4.0%)		
Pain type	1.38 (0.49)	1.63 (0.57)	< 0.001	< 0.001
Pain severity	2.66 (2.17)	4.03 (2.41)	< 0.001	< 0.001

^a^Mean (SD); *n* (%).

^b^Yuen's robust *t*-test; Fisher's exact test.

^c^False discovery rate correction for multiple testing.

The pattern of somatic symptoms was particularly striking, with mothers showing significantly higher levels (M = 1.39 vs. 0.94, *q* < 0.001). This aspect was accompanied by greater pain severity (M = 4.03 vs. 2.66, *q* < 0.001) and more diverse pain types (M = 1.63 vs. 1.38, *q* < 0.001) among mothers. These findings suggest that mothers may be experiencing and expressing their psychological distress through more pronounced physical symptoms than fathers.

Mothers also reported significantly higher intolerance of uncertainty about their son's or daughter's safety and the future (M = 2.80 vs. 2.50, *q* = 0.002) and utilized healthcare services more frequently (M = 2.34 vs. 2.15, *q* = 0.021), though the actual number of healthcare visits did not differ significantly between groups (*q* = 0.6). This pattern suggests that although both parents seek medical care at similar rates, mothers may require more intensive or repeated consultations when they do engage with healthcare services.

Interestingly, no significant differences were found between groups in their direct exposure to the October 7th events (*q* = 0.6), adverse life events (*q* = 0.2), medication use (*q* = 0.2), smoking (*q* = 0.2), or alcohol use (*q* > 0.9). The lack of differences in substance use is particularly noteworthy as it suggests that neither fathers nor mothers are disproportionately turning to these potentially maladaptive coping strategies, with alcohol use remaining remarkably low in both groups (4.5% fathers, 4.0% mothers).

### Network architecture

**Figure 1:**
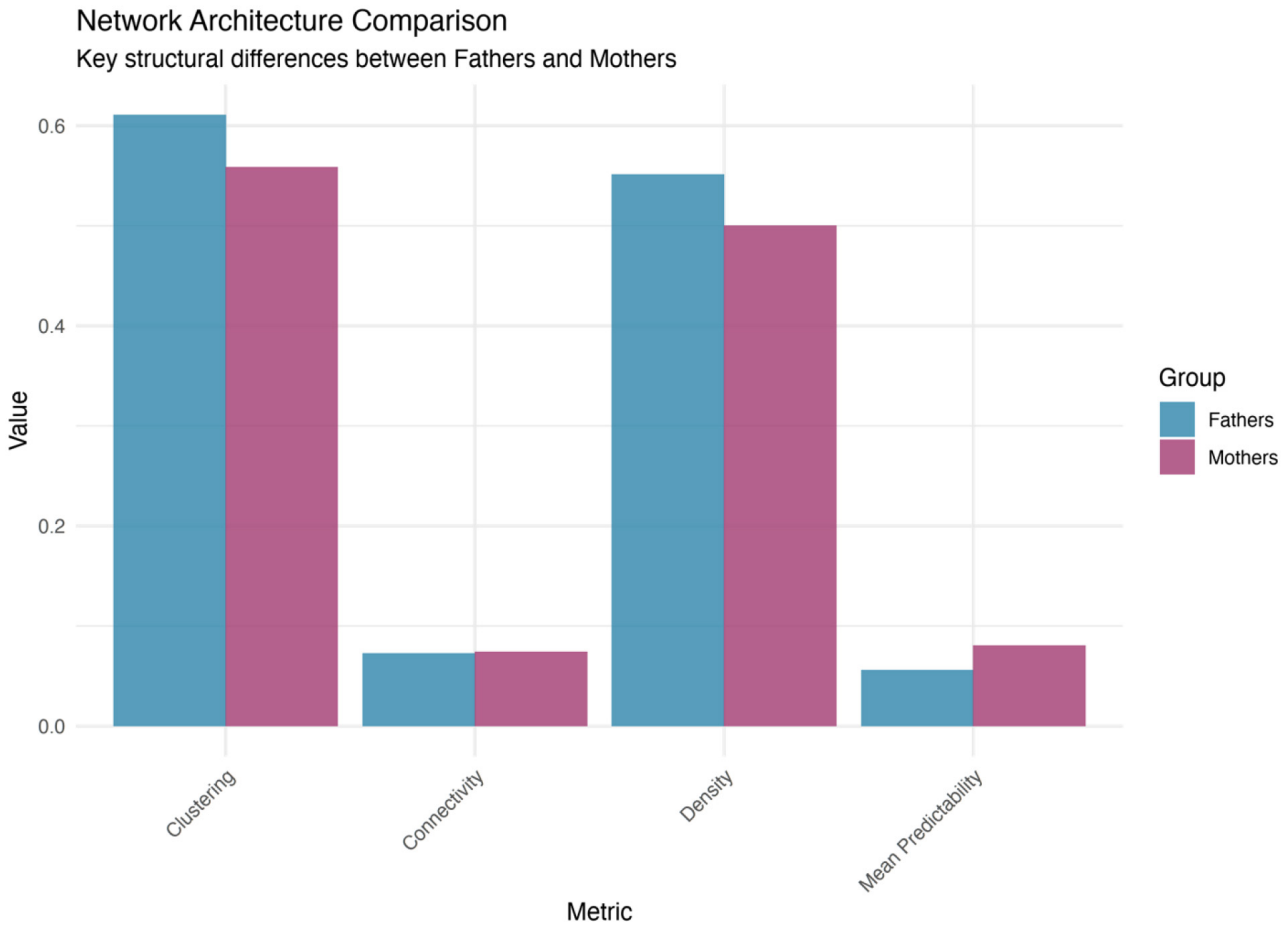
Network architecture comparison between fathers and mothers across four structural metrics. The clustering coefficient reflects the tendency for nodes to form tightly connected groups within the network. Connectivity represents the mean absolute edge weight across all connections. Density indicates the proportion of possible connections that are present in each network. Mean predictability shows the average proportion of variance in nodes explained by all other nodes in the network. Fathers demonstrate higher clustering (0.611 vs. 0.559) and density (0.551 vs. 0.500), while maintaining similar connectivity levels (0.072 vs. 0.074). Mothers show notably higher mean predictability (0.081 vs. 0.060), indicating more deterministic symptom patterns despite having fewer overall connections.

Network architecture analysis (see Figure 1) revealed both striking similarities and important differences in psychological distress, somatic symptoms, health behavior, and healthcare utilization among fathers and mothers. The connectivity measures, representing the mean absolute edge weights, were remarkably similar between groups (fathers: 0.072; mothers: 0.074), as were the global strength values (fathers: 5.65; mothers: 5.79), indicating that both parent groups showed comparable overall levels of interconnection in health domains (i.e., psychological, somatic, and behavioral alongside patterns of healthcare utilization). However, notable differences emerged in network density and organization. Fathers showed slightly higher network density (0.551) than did mothers (0.500), indicating that a greater proportion of possible connections were present in the fathers' network. This finding suggests that fathers' responses may be more broadly interconnected, with various symptoms and behaviors influencing each other in a more distributed pattern.

Small-world analysis revealed particularly intriguing differences in network topology. Fathers exhibited higher clustering coefficients (0.611) than did mothers (0.559), indicating stronger local organization where related symptoms and behaviors tended to cluster together in fathers' networks. Importantly, mothers showed substantially shorter average path lengths (9.44) than did fathers (11.82).

This finding, where mothers had shorter paths despite lower density, suggests fundamentally different organizational principles between the parent groups. In mothers' networks, psychological distress appeared to propagate more efficiently across different domains despite having fewer overall connections. The shorter path lengths in mothers' networks indicate that a change in one area (such as sleep disturbances) can more quickly influence distant areas (such as pain severity), creating a system where distress signals travel more rapidly between different symptom domains.

The combination of lower density but shorter path lengths in mothers' networks suggests a more streamlined architecture where specific, critical pathways connect different symptom clusters. In contrast, fathers' networks showed higher density with longer path lengths, indicating a more redundant system with multiple pathways that may provide more buffering against psychological distress propagation but require more steps for information to travel across the network.

### Network structure and communities

**Figure 2:**
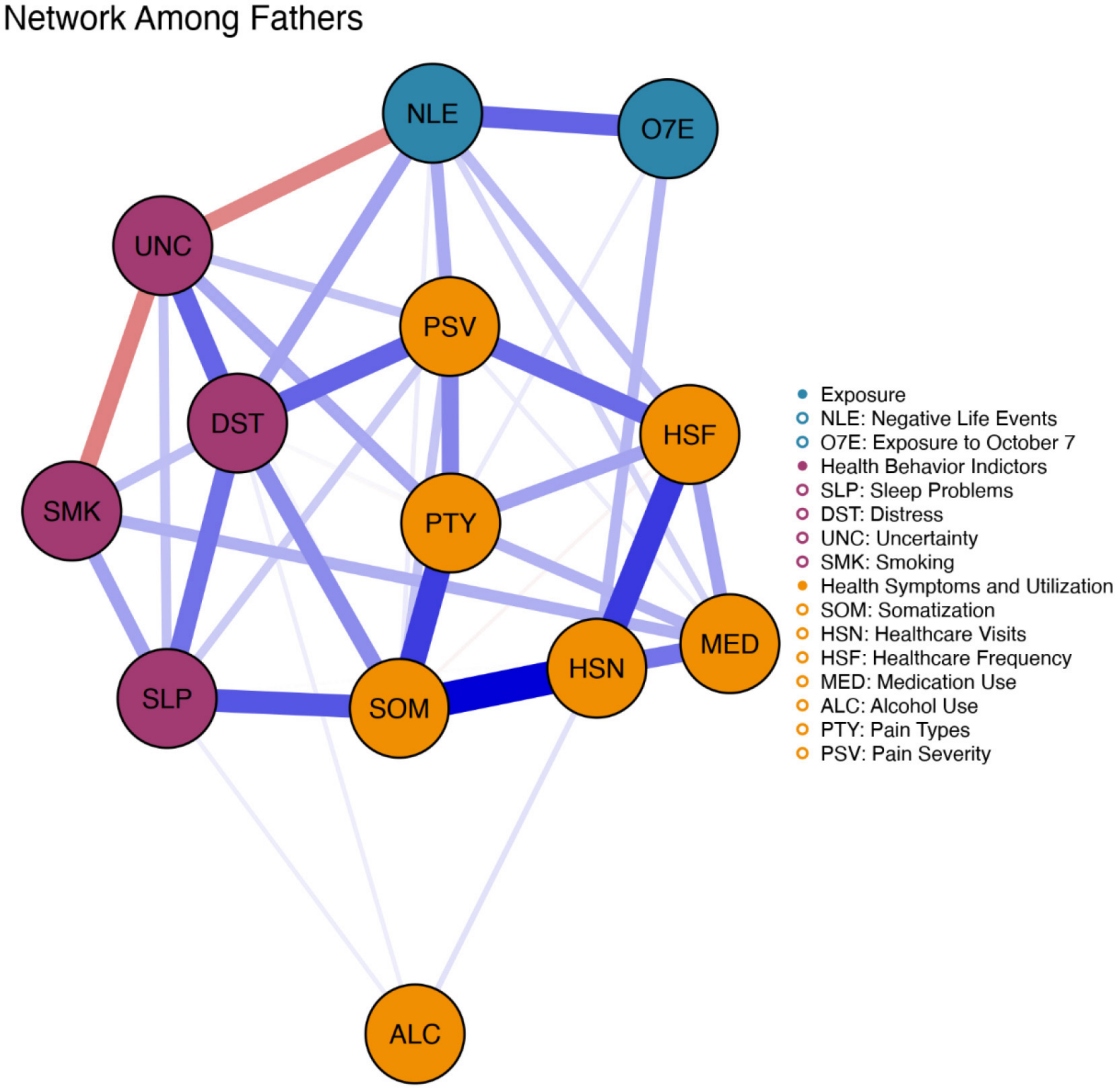
Network structure among fathers of offspring currently on active duty. Network visualization showing interconnections between psychological, behavioral, and somatic symptoms. Blue edges indicate positive associations, whereas red edges indicate negative associations. Edge thickness and proximity reflect connection strength. Nodes are color-coded by community: behavioral distress (teal/blue: sleep problems, distress, uncertainty, smoking), medical burden (orange/yellow: somatization, healthcare visits/frequency, medication use, pain type/severity, alcohol use), and exposure (purple: negative life events, October 7^th^ exposure).

**Figure 3:**
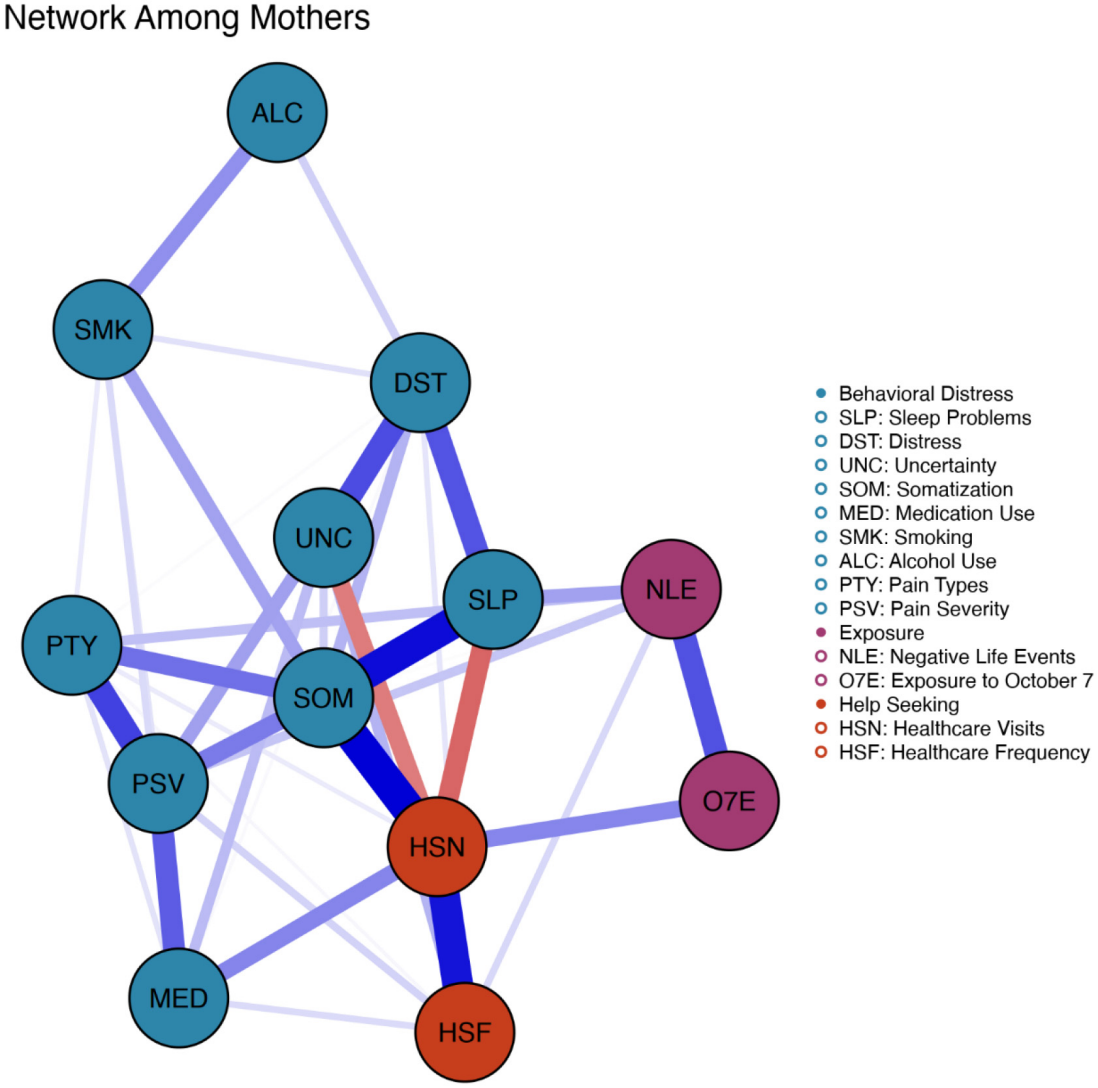
Network structure among mothers of offspring currently on active duty. Network visualization displaying interconnections between psychological, behavioral, and somatic symptoms. Blue edges indicate positive associations, whereas red edges indicate negative associations. Edge thickness and proximity reflect connection strength. Nodes are color-coded by community: behavioral distress (teal/blue: sleep problems, distress, intolerance of uncertainty, somatic symptoms, medication use, smoking, alcohol use, pain type/severity), help-seeking (orange/red: healthcare visits and frequency), and exposure (purple: Adverse life events, October 7th exposure).

The analysis revealed fundamentally different network organizations between fathers and mothers (see Figures 2, 3). Visual inspection of the network structures shows distinct patterns in how symptoms and behaviors clustered and interconnected between the two parent groups.

In the mothers' network, we observed three distinct communities: (1) a health behavior indictors cluster (shown in teal/blue) encompassing sleep disturbances, psychological distress, intolerance of uncertainty, somatic symptoms, pain type, pain severity, smoking, alcohol use, and medication use; (2) a healthcare utilization cluster (shown in orange/red) comprising healthcare visits and healthcare frequency; and (3) an exposure cluster (shown in purple) containing adverse life events and exposure to October 7th events. The healthcare utilization nodes serve as critical bridges between the health behavior indicators and exposure communities, with particularly strong connections between somatic symptoms and healthcare visits, indicating that physical manifestations of stress directly drove healthcare utilization in mothers.

In contrast, the fathers' network exhibited a more integrated but differentiated structure with four identifiable communities: (1) a health behavior indictors cluster (teal/blue) including sleep disturbances, psychological distress, intolerance of uncertainty, and smoking; (2) a health symptoms and utilization cluster (orange/yellow) encompassing somatic symptoms, pain severity, pain type, healthcare visits, healthcare frequency, and medication use; (3) an exposure cluster (purple) with adverse life events and exposure to October 7th; and (4) alcohol use appearing as a relatively isolated node, although statistically related to the health symptoms and utilization cluster. This organization suggests that fathers compartmentalized their responses more distinctly, with health symptoms and utilization forming a unified cluster separate from psychological distress.

A striking difference between the networks is the positioning and connectivity of key nodes. In mothers' networks, somatic symptoms occupied a more central position with stronger connections to both health behavior indicators and healthcare utilization. Further, the thick edges between psychological distress, somatic symptoms, and sleep disturbances in mothers suggest that these formed a tightly coupled triad of symptoms. In fathers' networks, these connections appeared more diffuse, with somatic symptoms more strongly connected to other health symptoms (pain) than to psychological distress.

The exposure-related nodes (adverse life events and October 7th exposure) show interesting patterns. In mothers, these nodes were more peripherally positioned but maintained connections to the main health behavior indicators through healthcare utilization nodes. In fathers, adverse life events appeared more centrally positioned with direct connections to multiple symptom domains, suggesting that external stressors may have had more direct and varied impacts on fathers' symptom networks.

Notably, substance use behaviors showed different integration patterns. In mothers' networks, smoking and alcohol use were embedded within the health behavior indicators cluster, suggesting that these behaviors were part of an integrated stress response. In fathers' networks, alcohol use appeared isolated at the network's periphery, whereas smoking maintained connections to the health behavior indicators cluster, indicating that fathers may have used these coping mechanisms more selectively or independently of other symptoms.

### Centrality invariance

**Figure 4:**
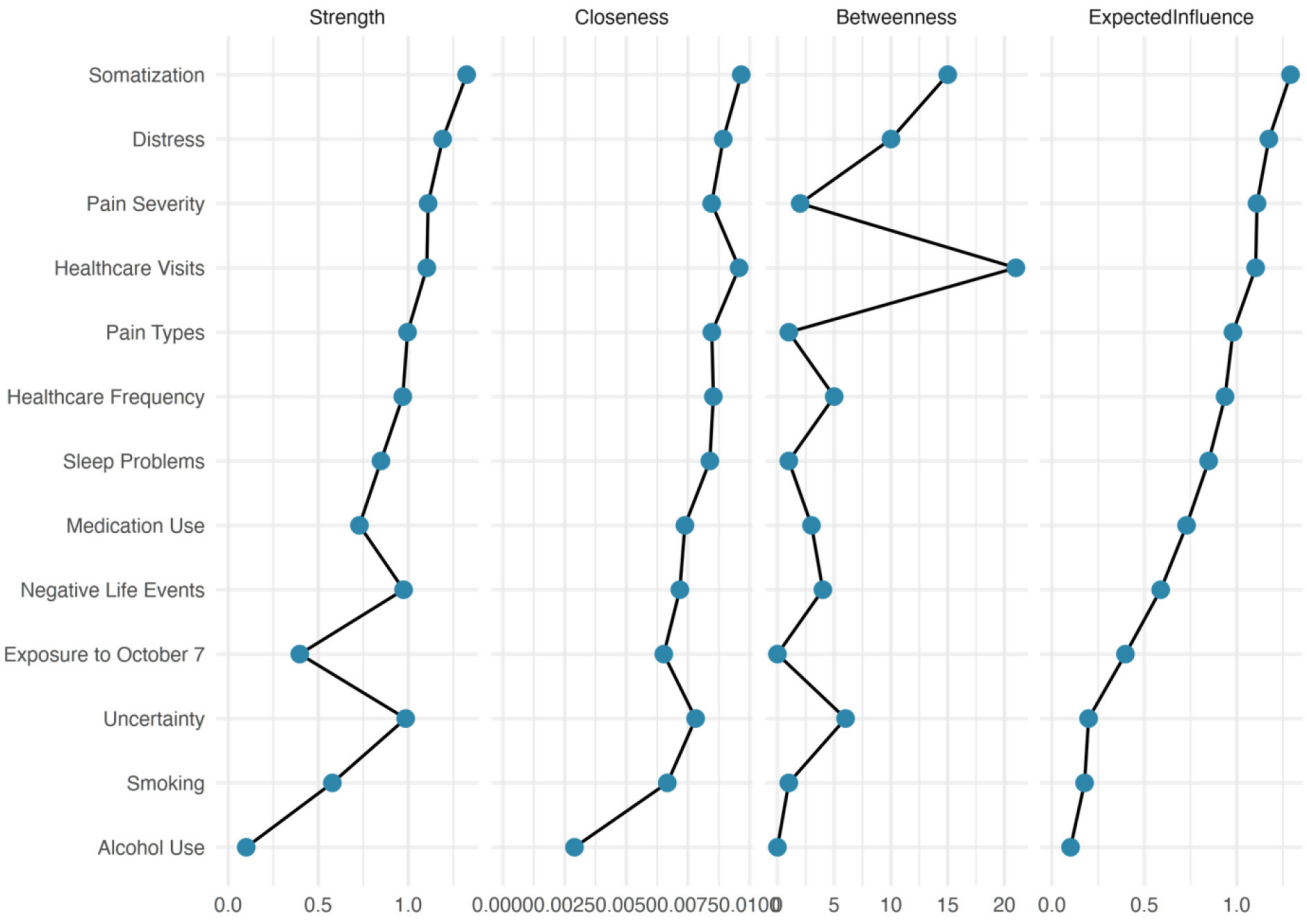
Centrality measures for the fathers' network. Four centrality indices are displayed for each variable in the network. Strength centrality (leftmost) represents the sum of absolute edge weights connected to each node. Closeness centrality measures how quickly activation can spread from a node to all other reachable nodes. Betweenness centrality quantifies how often a node lies on the shortest path between other node pairs, identifying potential bridges in the network. Expected influence accounts for both positive and negative connections, providing the net influence of each node.

**Figure 5:**
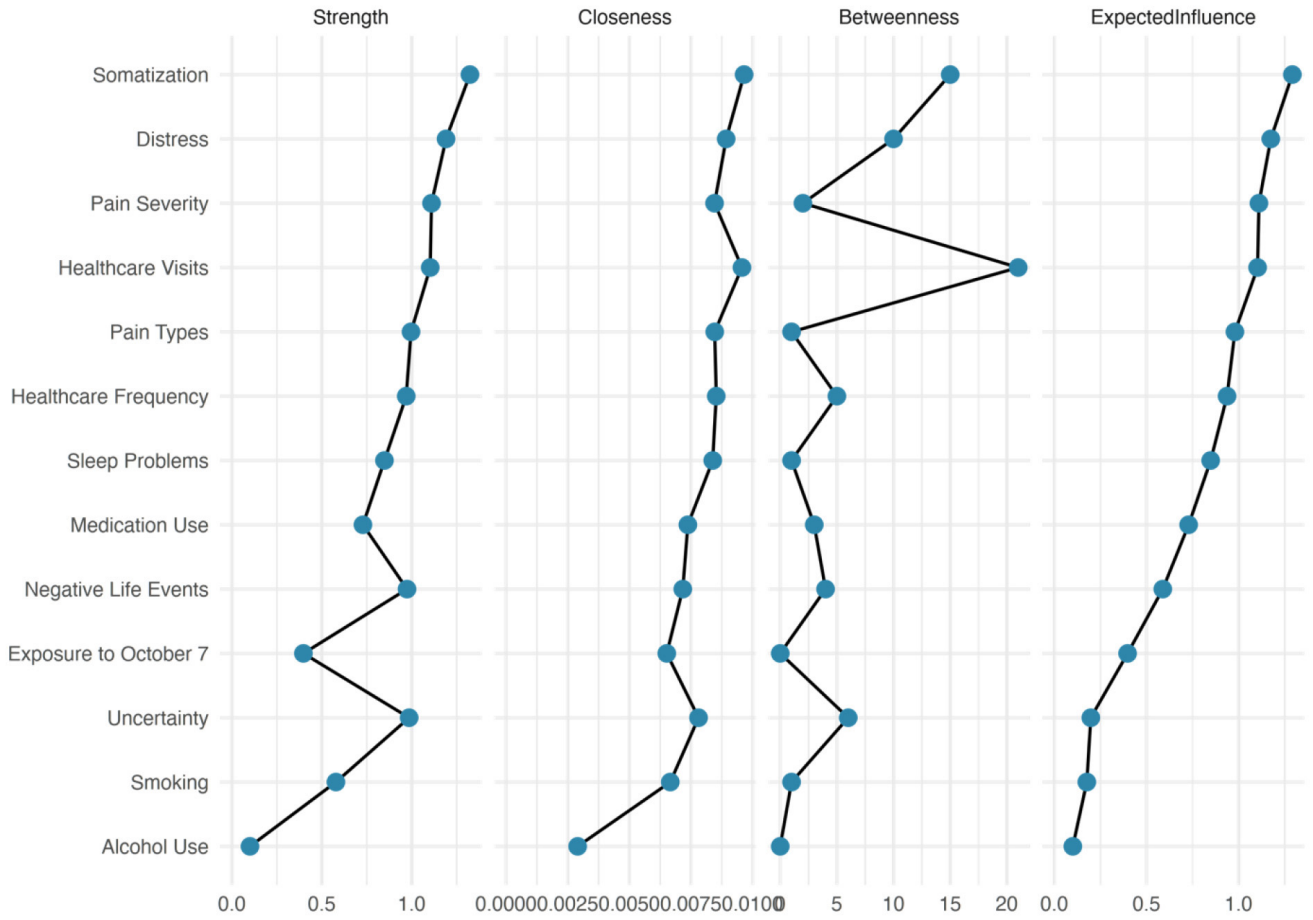
Centrality measures for the mothers' network. Four centrality indices are displayed for each variable in the network. Strength centrality (leftmost) represents the sum of absolute edge weights connected to each node. Closeness centrality measures how quickly activation can spread from a node to all other reachable nodes. Betweenness centrality quantifies how often a node lies on the shortest path between other node pairs, identifying potential bridges in the network. Expected influence accounts for both positive and negative connections, providing the net influence of each node.

As illustrated in Figures 4, 5, centrality invariance tests revealed significant differences between fathers and mothers in how certain variables functioned within their respective networks. After FDR correction, two key variables showed significant differences in centrality between groups. Specifically, somatic symptoms demonstrated significantly different closeness centrality between fathers and mothers (*p* = 0.058, *q* < 0.10), indicating that this variable occupied fundamentally different positions in the network architecture of each parent group. In mothers' networks, somatic symptoms appeared to have higher closeness centrality, meaning they could more quickly influence and be influenced by other symptoms in the network. This finding aligns with our earlier observations that mothers showed stronger integration between psychological distress and physical symptoms.

Sleep disturbances also showed marginally significant differences in expected influence centrality (*p* = 0.065, *q* < 0.10), suggesting that sleep disturbances may have had different net impacts on the overall network functioning between fathers and mothers. The higher expected influence in mothers' networks indicates that sleep disturbances may have served as a more powerful driver of other symptoms, potentially acting as an amplifier of distress that reverberated throughout the network.

All other centrality measures showed no significant differences between groups after FDR correction (all *q* > 0.10). These centrality measures include variables such as psychological distress, intolerance of uncertainty, healthcare utilization, pain severity, and substance use behaviors, which function similarly in terms of their network centrality despite the different overall network structures observed between fathers and mothers.

The centrality stability analysis revealed adequate stability for all centrality indices in both groups. Expected influence showed the highest stability across both networks, maintaining correlations above 0.90 even when only 30% of cases were sampled. Strength and closeness centrality also demonstrated good stability (correlations > 0.60 at 30% sampling), whereas betweenness centrality showed the lowest stability, particularly in fathers' networks, where it dropped below 0.40 at 30% sampling. This pattern suggests that although most centrality findings were robust, interpretations of betweenness centrality should be made with caution.

The stability differences between fathers and mothers were minimal, with mothers showing slightly better stability across most indices, possibly due to their more tightly coupled network structure. The consistent stability of expected influence and strength centrality in both groups indicates that our identification of key symptoms was reliable and not dependent on specific subsamples of participants.

### Bridge centrality analysis

**Figure 6:**
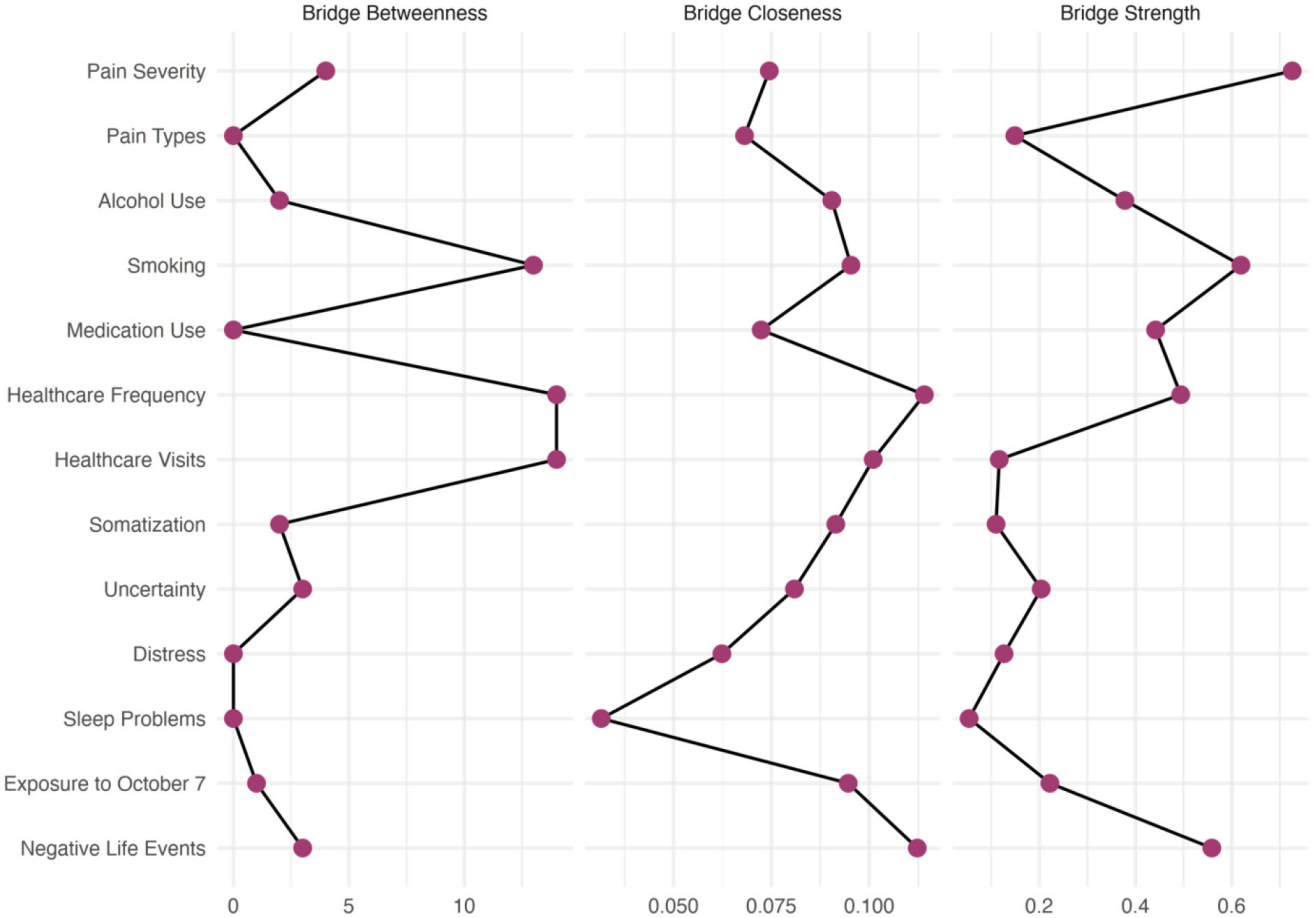
Bridge centrality measures for the fathers' network. Three bridge centrality indices identify nodes that connect different communities within the network. Bridge betweenness **(left)** quantifies how often a node lies on the shortest path between nodes in different communities. Bridge closeness **(center)** measures how quickly a node can reach nodes in other communities. Bridge strength **(right)** represents the sum of edge weights connecting a node to other communities.

**Figure 7:**
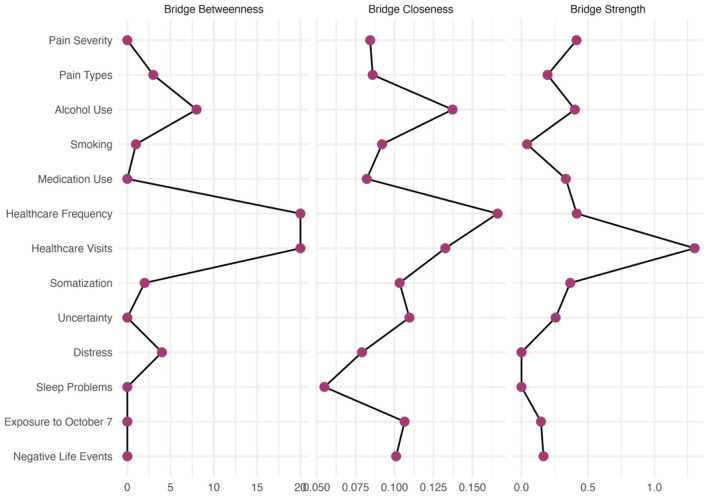
Bridge centrality measures for the mothers' network. Three bridge centrality indices identify nodes that connect different communities within the network. Bridge betweenness **(left)** quantifies how often a node lies on the shortest path between nodes in different communities. Bridge closeness **(center)** measures how quickly a node can reach nodes in other communities. Bridge strength **(right)** represents the sum of edge weights connecting a node to other communities.

Bridge centrality analysis (see Figures 6, 7) revealed critical differences in how fathers and mothers connected different health domains within their networks. In fathers' networks, somatic symptoms emerged as the most critical bridge node with the highest bridge closeness (0.114) and bridge betweenness (14), serving as the primary connector between health behavior indicators and health symptoms and utilization communities. Pain severity also showed high bridge strength (0.559), indicating strong connections across communities. Notably, psychological distress demonstrated the second-highest bridge betweenness (13), suggesting that it frequently mediated pathways between different symptom domains. Adverse life events showed substantial bridge strength (0.726), connecting exposure experiences to both health behavior indicators and healthcare utilization domains.

Mothers' networks revealed a strikingly different pattern. Healthcare visits dominated as the supreme bridge node with exceptionally high bridge strength (1.302), more than double any other variable, and the highest bridge betweenness (20). This finding indicates that healthcare utilization served as the critical junction between the large health behavior indicators community and healthcare utilization in mothers. Somatic symptoms, although still important (bridge strength = 0.416, bridge betweenness = 20), functioned differently than in fathers: They bridged within the health behavior indicators community rather than between distinct communities.

The different role of healthcare utilization is particularly noteworthy. In mothers, healthcare visits served as the primary bridge with a bridge strength of 1.302, whereas in fathers, this value was only 0.117. This 10-fold difference suggests that mothers' healthcare utilization was much more tightly integrated with their symptom experiences, potentially explaining their higher healthcare utilization frequency.

Sleep disturbances showed interesting bridging patterns, with comparable bridge strength in both groups (fathers: 0.378; mothers: 0.401) but higher bridge betweenness in mothers (8 vs. 2), suggesting that it connected more diverse symptom domains in mothers' networks. Conversely, intolerance of uncertainty showed higher bridging in fathers (0.442) than in mothers (0.333), indicating that it played a more connective role in fathers' symptom networks.

Substance use behaviors showed minimal bridging functions in both groups, with smoking and alcohol use showing near-zero bridge strength in mothers' networks, suggesting that these behaviors were isolated within their respective communities rather than connecting different symptom domains.

Given the above, the simpler community structure in mothers with fewer but stronger bridges indicates a more vulnerable network architecture; disruption of key bridges, such as healthcare visits, could isolate entire health behavior indicators and symptoms. In contrast, fathers' more complex community structure with multiple moderate bridges suggests greater network resilience.

### Edges invariance

Edge invariance testing revealed four edges that differed significantly between fathers' and mothers' networks at the nominal significance level (*p* < 0.05), though none survived FDR correction (all *q* > 0.10; see Table 3). Visual inspection of the networks combined with statistical testing reveals important patterns in how symptoms interconnected differently between parent groups.

**Table 3:** Edge invariance test.

Variable 1	Variable 2	*p* ^a^
Sleep disturbances	Smoking	**0.004**
Medication use	Pain severity	**0.016**
Adverse life events	Sleep disturbances	**0.040**
Intolerance of uncertainty	Pain type	**0.047**
Intolerance of uncertainty	Pain severity	0.056
Adverse life events	Psychological distress	0.085
Somatic symptoms	Healthcare frequency	0.094
Healthcare frequency	Pain severity	0.099
Intolerance of uncertainty	Medication use	0.100
Intolerance of uncertainty	Healthcare frequency	0.109
Psychological distress	Healthcare frequency	0.110
Sleep disturbances	Intolerance of uncertainty	0.115
Somatic symptoms	Smoking	0.120
Adverse life events	Intolerance of uncertainty	0.126
Healthcare visits	Pain type	0.165
Adverse life events	Medication use	0.168
Healthcare frequency	Pain type	0.202
Pain type	Pain severity	0.208
Intolerance of uncertainty	Healthcare visits	0.215
Somatic Symptoms	Pain severity	0.251
Exposure to October 7th	Pain type	0.267
Sleep disturbances	Somatic symptoms	0.293
Sleep disturbances	Pain severity	0.305
Psychological distress	Pain severity	0.314
Sleep disturbances	Alcohol use	0.317
Smoking	Alcohol use	0.354
Somatic symptoms	Pain type	0.398
Sleep disturbances	Healthcare visits	0.432
Exposure to October 7th	Healthcare visits	0.453
Intolerance of uncertainty	Smoking	0.460
Healthcare visits	Healthcare frequency	0.481
Psychological distress	Somatic symptoms	0.485
Medication use	Pain type	0.492
Healthcare frequency	Medication use	0.526
Sleep disturbances	Psychological distress	0.555
Medication use	Smoking	0.562
Intolerance of uncertainty	Somatic symptoms	0.563
Adverse life events	Pain type	0.564
Psychological distress	Intolerance of uncertainty	0.635
Sleep disturbances	Medication use	0.636
Adverse life events	Pain severity	0.645
Smoking	Pain severity	0.647
Adverse life events	Healthcare frequency	0.678
Smoking	Pain type	0.679
Psychological distress	Smoking	0.700
Psychological distress	Alcohol use	0.711
Adverse life events	Somatic symptoms	0.713
Healthcare visits	Alcohol use	0.742
Adverse life events	Exposure to October 7th	0.759
Psychological distress	Pain type	0.791
Somatic symptoms	Medication use	0.817
Healthcare visits	Medication use	0.820
Somatic symptoms	Healthcare visits	0.936
Psychological distress	Medication use	0.940
Exposure to October 7th	Sleep disturbances	1.000
Exposure to October 7th	Psychological distress	1.000
Exposure to October 7th	Intolerance of uncertainty	1.000
Exposure to October 7th	Somatic symptoms	1.000
Adverse life events	Healthcare visits	1.000
Psychological distress	Healthcare visits	1.000
Exposure to October 7th	Healthcare frequency	1.000
Sleep disturbances	Healthcare frequency	1.000
Exposure to October 7th	Medication use	1.000
Adverse life events	Smoking	1.000
Exposure to October 7th	Smoking	1.000
Healthcare visits	Smoking	1.000
Healthcare frequency	Smoking	1.000
Adverse life events	Alcohol use	1.000
Exposure to October 7th	Alcohol use	1.000
Intolerance of uncertainty	Alcohol use	1.000
Somatic symptoms	Alcohol use	1.000
Healthcare frequency	Alcohol use	1.000
Medication use	Alcohol use	1.000
Sleep disturbances	Pain type	1.000
Alcohol use	Pain type	1.000
Exposure to October 7th	Pain severity	1.000
Healthcare visits	Pain severity	1.000
Alcohol use	Pain severity	1.000

^a^Bolded *p*-values are nominally significant at *p* < 0.05.

The strongest difference was found in the connection between sleep disturbances and smoking (*p* = 0.004). This edge appeared stronger in mothers' networks, where it showed as a more prominent connection. This finding indicates that for mothers, sleep disturbances and smoking behaviors were more tightly coupled, potentially suggesting that mothers may have been more likely to use smoking as a coping mechanism for sleep disturbances or that smoking more directly impacted their sleep quality.

The edge between medication use and pain severity showed a significant difference (*p* = 0.016). This connection appeared stronger in fathers' networks, suggesting that fathers showed a more direct relation between pain intensity and medication utilization. This finding could reflect different thresholds or patterns in how fathers translate pain experiences into pharmaceutical utilization.

The connection between adverse life events and sleep disturbances differed significantly (*p* = 0.040). This edge was more prominent in mothers' networks, indicating that external life stressors may have had more direct impacts on mothers' sleep quality. Finally, the edge between intolerance of uncertainty and pain type showed significance (*p* = 0.047). This connection was stronger in fathers, indicating that intolerance of uncertainty may have translated more directly into diverse pain experiences in fathers. The fact that most edges showed no significant differences despite different symptom levels and network architectures suggests that many fundamental symptom relations remained consistent across both parent populations.

### Edge weight stability

Bootstrap analyses with 1,000 iterations examined the stability of edge weights in both networks, revealing adequate stability with interesting differences in reliability patterns. Fathers' networks contained 43 non-zero edges out of 78 possible connections, representing 55.1% density, whereas mothers' networks showed 39 non-zero edges at 50.0% density. Despite this difference in the number of connections, both networks maintained remarkably similar mean edge weights of 0.062, suggesting that the intensity of existing connections remained comparable even as their distribution differed.

Visual inspection of the stability plots reveals that the strongest edges in both networks demonstrated the narrowest confidence intervals, indicating high reliability in their estimation. The somatic symptoms and healthcare visits connection emerged as one of the most stable edges across both parent groups, consistently maintaining its strength across bootstrapped samples. This stability is particularly noteworthy given the clinical importance of this connection in understanding how physical symptoms translate into healthcare utilization.

Mothers' networks demonstrated somewhat tighter confidence intervals overall, particularly for edges in the lower portion of the distribution, suggesting more consistent estimation of weaker connections. The somatization-healthcare frequency connection showed consistent positive values across resamples, whereas the relation between sleep disturbances and healthcare visits maintained a stable pattern throughout the bootstrap procedure. The connection between adverse life events and intolerance of uncertainty also demonstrated remarkable consistency in mothers' networks.

Fathers' networks displayed wider confidence intervals for several edges, particularly those in the middle range of the distribution, though certain connections showed exceptional stability. The somatization-healthcare visits edge emerged as the strongest and most stable positive connection, whereas the sleep disturbances-healthcare visits relation showed a different pattern than in mothers, maintaining its distinctive character across resamples. The intolerance of uncertainty-healthcare frequency connection, although more variable, remained consistently present throughout the bootstrap samples.

### Predictability analysis

Node predictability analysis revealed significant differences in how deterministic the health symptoms and healthcare utilization networks were between fathers and mothers. All 13 variables in mothers' networks showed predictability values greater than zero, whereas 12 out of 13 variables in fathers' networks demonstrated non-zero predictability, with alcohol use being completely unpredictable from other network variables in fathers. This fundamental difference suggests that mothers' health symptoms and healthcare utilization networks operate as more fully integrated systems where every element is influenced by the network structure.

The mean predictability among non-zero values was significantly higher in mothers than in fathers, with mothers showing a mean of 0.081 vs. fathers' 0.060. This difference was statistically significant according to the Mann-Whitney U test (*p* = 0.026) and represented a large effect size (Cohen's d = 1.04). This substantial difference indicates that mothers' health symptoms and healthcare utilization were more tightly determined by their network structure, suggesting less flexibility but potentially more predictable intervention effects.

The most striking difference emerged in alcohol use predictability, which showed zero predictability in fathers but 0.084 in mothers. This finding suggests that fathers' alcohol use operated independently of their other symptoms and stressors, potentially reflecting habitual or social patterns rather than stress-responsive drinking. In contrast, mothers' alcohol use appeared integrated into their symptom network, potentially serving as a coping mechanism that responded to other network elements.

Intolerance of uncertainty showed markedly higher predictability in mothers than in fathers, with values of 0.114 vs. 0.050, respectively. This finding indicates that mothers' intolerance of uncertainty was more strongly determined by other health behavior indicators in the network. Healthcare frequency also demonstrated higher predictability in mothers, suggesting that their healthcare utilization patterns were more directly driven by their health behavior indicators than being independent help-seeking decisions.

Smoking behavior showed interesting patterns, with predictability values of 0.082 in mothers vs. 0.036 in fathers. This finding aligns with the edge invariance findings, confirming that mothers' smoking was more tightly coupled with their overall health behavior indicators network, particularly with sleep disturbances and psychological distress. Higher predictability suggests that addressing other network elements could more effectively influence smoking behavior in mothers than in fathers.

Pain-related variables showed relatively similar predictability between groups, with pain severity slightly more predictable in mothers and pain types showing nearly identical values. This finding suggests that although mothers experienced more severe pain overall, the fundamental mechanisms determining pain experiences may have been similar across both parent groups. Somatic symptoms demonstrated comparable predictability in both networks, indicating that the translation of psychological distress into physical symptoms follows similar network-determined patterns regardless of gender.

Sleep disturbances and healthcare visits showed the highest predictability values in both groups, exceeding 0.097 in fathers and 0.103 in mothers. These high values indicate that sleep disturbances and healthcare utilization were strongly determined by network structure in both parent populations, making them potentially reliable intervention targets where changes in connected symptoms would predictably influence these outcomes.

The exposure-related events showed moderate predictability in both groups, with exposure to October 7th events slightly more predictable in mothers, and adverse life events showing similar values. This finding suggests that how parents process and integrate traumatic experiences is partially determined by their existing symptom patterns, though substantial variance remains unexplained by network structure alone.

## Discussion

In the present study, we examined how fathers and mothers of soldiers (regular or reserve) serving during a war differed in health domains (i.e., psychological, somatic, and behavioral alongside patterns of healthcare utilization). By applying robust statistical techniques and network analysis, we revealed a striking gender divide: Whereas both fathers and mothers were exposed to similar external stressors associated with their child's active military service, mothers reported substantially higher levels of psychological distress, sleep disturbances, somatic symptoms, and pain (severity and type), alongside more frequent engagement with healthcare services. Network analyses further underscored these differences, showing that mothers' responses were more tightly interconnected, with somatic symptoms and healthcare utilization occupying central bridging roles, whereas fathers displayed more compartmentalized networks with broader but less efficient interconnections. Taken together, these results highlight the disproportionate burden experienced by mothers, who seem to manifest their distress more somatically and engage in help-seeking behaviors that are closely intertwined with their symptom profiles.

Indeed, accumulating evidence demonstrates robust gender differences in both somatic symptom reporting and help-seeking behaviors. For example, in veteran samples, women reported more severe somatic symptoms and higher rates of post-traumatic stress disorder (PTSD) and pain complaints than men (33). Similarly, van den Houdt et al. (34) showed that women and individuals who identified with more feminine norms reported higher somatic complaints than their male or more masculine-norm counterparts. Moreover, Ballering et al. (35) found that the female sex, rather than traits of femininity *per se*, was strongly associated with higher primary care help-seeking for new-onset somatic symptoms. In addition, their longitudinal data showed that women were more likely to consult a general practitioner for somatic complaints than were men. These differences are further intensified by exposure to external stressors associated with active military service. Women, compared to men, report a higher symptom burden and greater functional impairment, as well as a stronger tendency to disclose distress and seek care (36, 37).

A possible explanation for our findings may derive from biopsychosocial stress processes. Biologically, sex-specific genetic and neuroendocrine mechanisms contribute to differences in stress responses and in how symptoms are perceived. For example, women exhibit higher methylation levels of the serotonin transporter gene (SLC6A4), combined with hormonal fluctuations, which seem to increase interoceptive sensitivity, leading them to notice and report bodily sensations when they are under stress (38, 39). Because mothers often carry greater caregiving burdens, their physiological load is likely higher; this higher load, in turn, may increase their somatic symptom burden and drive higher health service utilization.

Another potential explanation can be found in social role theory (40), which holds that cultural norms and societal expectations strongly influence how men and women handle emotional expression and stress. Women are generally socialized to recognize emotional distress and are encouraged to seek help, potentially resulting in higher reported psychological symptoms. In contrast, men are more often socialized to conceal vulnerability, potentially leading to an underreporting of distress. In line with this notion, Levi-Belz et al. (41) conducted a longitudinal study of Israeli citizens in the immediate aftermath of the October 7th, 2023, terrorist attack. They found that women showed greater vulnerability to PTSD, depression, and anxiety after the attack, with symptom increases from before to after the attack that went beyond demographic and direct exposure factors. Moreover, the “tend-and-befriend” model (42) suggests that women respond to stress by seeking social support and sharing their distress, whereas men more often rely on fight-or-flight responses (43). These gendered patterns influence differences in symptom expression and in care-seeking. Taken together, biological and sociocultural frameworks help explain our observed pattern: Under conditions of military threat, mothers not only experienced greater distress but also tended to express it more somatically and seek help more often, reflected in denser symptom networks. Fathers, even when distressed, may report fewer somatic symptoms or seek help less, or do so in less structured or visible ways, weakening the connections in their networks.

Beyond these gendered differences in psychological distress, the network analyses provide additional insights. Network architecture differences showed that mothers' networks, though less dense, had shorter paths, implying faster spread of distress across symptoms. Fathers' networks, by contrast, were more compartmentalized, perhaps reducing amplified vulnerability. Community structure diverged further: In mothers, healthcare utilization acted as a bridge between psychological distress and exposure-related events, integrating somatic and help-seeking responses. For fathers, the somatic and psychological symptoms remained more separated, reflecting more isolated distress expression. In addition, centrality and bridge analyses indicated that somatic symptoms and sleep disturbances emerged as key connectors in mothers' networks, with healthcare visits acting as dominant bridges. In fathers, bridges were stronger around adverse life events and pain. Finally, predictability analyses showed that mothers' networks were more deterministic (i.e., nodes were more predictable from other nodes), heightening both risks of amplified vulnerability and opportunities for targeted intervention; fathers' networks were less predictable, with behavioral nodes (e.g., alcohol use) more isolated. These findings suggest that mothers whose son or daughter is in military service show more integrated symptom networks, where somatic complaints and help-seeking are central hubs, whereas fathers exhibit more segmented networks, with distress more tied to pain but with less visible health service engagement.

The divergent health indicators network structures observed between mothers and fathers reflect not only different psychological responses to their offspring's military service during war, but also the deeply gendered ways in which psychological burden is embodied, expressed, and managed. Specifically, mothers' networks, characterized by shorter paths, higher predictability, and centralized nodes such as somatic symptoms and sleep disturbances, suggest a tightly coupled system in which distress spreads efficiently across psychological, physical, and behavioral domains. This configuration likely reflects gendered socialization processes that encourage emotional awareness, somatic awareness, and proactive help-seeking, behaviors reinforced by societal norms that position women as both emotionally expressive and health-attentive (44, 45). In contrast, fathers' networks appear more segmented, with lower predictability and more peripheral behavioral nodes such as alcohol use. These patterns align with normative masculinity scripts emphasizing emotional restraint, compartmentalization, and reluctance to seek help (46, 47), which may lead to more diffuse or externally modulated symptom configurations. Notably, although mothers' integrated networks may increase their risk of experiencing escalating distress, they also offer clear, centralized points for intervention, particularly within healthcare settings. Fathers' more fragmented networks may offer some insulation from rapid symptom escalation, but they risk obscuring distress and delaying support. These findings underscore how gender operates not only as a social category but as a structuring principle of psychological organization under threat, shaping both the architecture and accessibility of coping resources.

### Limitations

Several limitations should be noted. First, the study's cross-sectional design restricts causal inference as we cannot determine the directionality of associations among the study variables or how symptom networks evolve across time. Second, the sample may have been subject to selection bias and to social desirability; mothers and fathers who responded may have differed systematically (in terms of distress, help-seeking, or health behaviors) from those who did not, which could have influenced network structure and centrality findings. Moreover, the study relied exclusively on self-reported information, which reflects participants' subjective perceptions rather than objective health indicators. In addition, the nature of self-report questionnaires may lead to underreporting or misreporting, especially for fathers, due to social desirability or stigmatization of emotional expression (48, 49). Future research could benefit from incorporating qualitative interviews to capture more nuanced and dynamic aspects of being a parent of a soldier during a war. Third, cultural, contextual, and setting specificity may limit generalizability. The findings may reflect local gender norms, military culture, health system structure, or crisis context, which may differ in other sociocultural or national contexts. Fourth, the reliance on a convenience sample recruited through an online panel may limit the generalizability of findings to more diverse or underrepresented populations. Future studies should strive for broader representation across ethnic, cultural, and socioeconomic groups to better understand disparities in health indicators in general and during a war in particular. Likewise, the lack of detailed information regarding participants' access to social and community resources requires caution in interpreting observed gender differences in health and healthcare utilization. Future studies are recommended to include such resources to gain additional information that may account for disparities in health indicators among mothers and fathers of soldiers.

## Conclusions

In this study, we examined how mothers and fathers of actively serving offspring during a war differed in the organization of somatic, psychological, behavioral, and healthcare-utilization indicators, exploring how health domains were interrelated by gender and what these network architectures imply for theory and practice. Mothers reported higher psychological distress, sleep disturbances, somatic symptoms, and pain, and they engaged more frequently with healthcare services. Network analyses indicated that mothers' symptom networks were more tightly integrated, with shorter path lengths and higher predictability, such that changes in one domain (e.g., sleep disturbances) more quickly spread to others, including pain, somatic symptoms, and help-seeking. Healthcare visits operated as a dominant bridge linking symptoms to service use. In contrast, fathers' networks were denser yet more compartmentalized. Psychological distress was segmented, pain and adverse events served as key connectors, alcohol use was peripheral and less predictable, and help-seeking was less central. These patterns may reflect combined effects of biological stress sensitivity, gendered socialization, and structural pathways into care.

The study advances gender scholarship by demonstrating that mothers' networks are not only higher in symptom levels but also differently structured (more integrated and faster propagating), thus extending accounts of gendered embodiment under threat. The study also fills a gap in feminist health scholarship on caregiving in a military context by identifying leverage points and structural vulnerabilities (i.e., reliance on healthcare as a bridge), showing how health systems become gendered conduits of coping. Further, the study provides nuance regarding help-seeking narratives, as fathers' segmented networks suggest not an absence of distress but architectures that externalize or obscure it, complicating assumptions that lower male help-seeking signals lower need.

These gendered network differences carry clear implications. Measurement should assess gendered interrelations rather than symptom levels alone. For mothers, targeting central hubs (somatic symptoms, sleep disturbances, and help-seeking) may yield system-wide benefits. Beyond formal healthcare engagement, these findings also point to the potential relevance of somatic-oriented supportive and preventive practices. Approaches such as physical activity, body-based stress regulation, sleep-focused interventions, and other embodied strategies may represent accessible pathways for moderating somatic symptom burden and slowing the rapid spread of distress observed in mothers' networks. Although such practices were not directly assessed in the present study, they warrant consideration in future research and intervention frameworks addressing prolonged military-related stress. For fathers, reducing masculinity-related barriers and offering engagement pathways that validate behavioral expressions such as pain and substance use as legitimate entry points is essential. Finally, at policy and systems levels, services should accommodate these differences, legitimize men's emotional expression, expand supports systems beyond formal clinics, and challenge dominant masculine norms to normalize men's mental health engagement and recognize behavioral nodes as valid distress signals.

In sum, gender shapes not only the level of distress but also its organization, spread, and visibility to care systems. By identifying network-specific bridges and hubs, the study offers actionable targets for gender-sensitive screening and intervention and shows how institutions and cultural norms co-produce gendered health architectures under military-related stress, while underscoring the need for longitudinal and mixed-methods research across diverse settings to test causal pathways, track network changes, and refine contextually grounded, gender-responsive interventions.

## Data Availability

The datasets analyzed during the current study are available from the corresponding author upon reasonable request.
